# Efficacy and safety of neostigmine on treating gastrointestinal dysmotility in severe acute pancreatitis patients: study protocol for a randomized controlled trial

**DOI:** 10.1186/s13063-023-07086-6

**Published:** 2023-02-06

**Authors:** Han Sun, Yaqi Sheng, Tiekuan Du, Huadong Zhu

**Affiliations:** Emergency Department, State Key Laboratory of Complex Severe and Rare Diseases, Dongcheng District, Peking Union Medical College Hospital, Chinese Academy of Medical Science and Peking Union Medical College, No.1 Shuaifuyuan, Beijing, 100730 China

**Keywords:** Neostigmine, Severe acute pancreatitis, Gastrointestinal dysmotility, Randomized controlled trial

## Abstract

**Background:**

Acute pancreatitis is a serious threat to human health and gastrointestinal dysmotility is a common complication for acute pancreatitis patients, resulting in delayed feeding, oral feeding intolerance, paralytic ileus, and abdominal compartment syndrome. Currently, there are limited treatment for this complication.

Neostigmine is known to increase gastrointestinal motility and has been used to treat gastrointestinal dysmotility after surgery. However, research in treating acute pancreatitis with neostigmine is currently limited.

**Methods:**

This trial is a randomized, placebo-controlled, double-blinded, mono-centric trial that will test the hypothesis that neostigmine can improve gastrointestinal motility in patients with severe acute pancreatitis. Up to 56 patients will be randomized in this study receiving 0.5 mg/1 ml of neostigmine methylsulfate injection twice per day or 1 ml of saline injection twice per day. Defection time (aim 1), mortality and organ failure (aim 2), borborygmus, starting of enteral nutrition and intra-abdominal pressure (aim 3), and length of ICU and hospital stay (aim 4) will be assessed.

**Discussion:**

Findings from this study will provide data supporting the usage of neostigmine for treating severe acute pancreatitis patients with gastrointestinal dysmotility.

**Trial registration:**

This study is registered on chictr.org.cn with the identifier as ChiCTR2200058305. Registered on April 5, 2022.

## Administrative information

Note: the numbers in curly brackets in this protocol refer to SPIRIT checklist item numbers. The order of the items has been modified to group similar items (see http://www.equator-network.org/reporting-guidelines/spirit-2013-statement-defining-standard-protocol-items-for-clinical-trials/).

**Table:** 

Title {1}	Efficacy and safety of neostigmine on treating gastrointestinal dysmotility in severe acute pancreatitis patients: study protocol for a randomized controlled trial.
Trial registration {2a and 2b}	This study is registered on chictr.org.cn with the identifier as ChiCTR2200058305. Registered on April 5, 2022.
Protocol version {3}	This is version 1.2 of the protocol date March 28, 2022
Funding {4}	This study is unfunded, but internally sponsored by the emergency department of Peking Union Medical College Hospital
Author details {5a}	Emergency Department, State Key Laboratory of Complex Severe and Rare Diseases, Peking Union Medical College Hospital, Chinese Academy of Medical Science and Peking Union Medical College, Beijing, China.
Name and contact information for the trial sponsor {5b}	This trial is investigator initiated and funded internally by the emergency department of Peking Union Medical College Hospital. There is no industry sponsor.
Role of sponsor {5c}	This trial is funded internally by the emergency department of Peking Union Medical College Hospital. There is no industry sponsor. The funders reviewed previous versions of the proposal and submitted suggestions which were incorporated into final protocol. There is an Institutional Review Board (IRB) to monitor the study. The IRB will not have authority over the writing of the report nor the decision to submit it for publication.

## Introduction

### Background and rationale {6a}

Acute pancreatitis (AP) is a seriousthreat to human health globally. It is the third most common gastrointestinal, liver, and pancreatic principal diagnoses from hospital admissions in USA. Over 275,000 patients were admitted because of AP every year, with over 2000 deaths and 2,600,000,000 dollars spent [1–3]. The diagnosis of AP requires 2 of the following features: characteristic abdominal pain, biochemical evidence of pancreatitis, radiographic evidence of pancreatitis. AP can be categorized as mild, moderately severe, or severe based on the revised Atlanta classification [4]. Severe acute pancreatitis (SAP), defined as pancreatitis with persist organ failure (over 48 h), develops in 20–30% of AP patients, with high mortality (15%) [5, 6].

Gastrointestinal dysmotility is a common complication for AP patients, often resulting in abdominal distension, nausea, and vomiting. Delayed feeding and oral feeding intolerance (OFI) may also be associated with gastrointestinal dysmotility. Severe gastrointestinal dysmotility is associated with paralytic ileus and abdominal compartment syndrome (ACS), which can be lethal for SAP patients [7–9]. Currently, there is limited evidence for treating gastrointestinal dysmotility in AP patients.

Early enteral nutrition (EN) plays an important role in the management of AP by protecting the mucosal barrier and reducing bacterial translocation. Delayed feeding (generally defined as over 24 h after onset) is associated with higher rates of infected peripancreatic necrosis, multiple organ failure, and total necrotizing pancreatitis [10–14]. OFI is a well-known complication of AP and the burden can be high. OFI develops in nearly one in six patients with AP, leading to longer length of hospitalization and reduced quality of life [15–19]. ACS, defined by the increased intra-abdominal pressure > 20 mmHg in association with failure of at least one organ system, developed in 12% to 56% of patients and the mortality of SAP patients with ACS is around 24 to 75% [20–24].

Neostigmine, an acetylcholinesterase inhibitor (ACI), is known to increase upper and lower gastrointestinal motility [25–27]. It has been used for postoperative recovery of gastrointestinal function [28, 29]. One study have shown that neostigmine could reduce intra-abdominal pressure (IAP) in SAP patients [30]. however, this is a single-center study with small sample size and no blinding was used. Further evidence is needed and this study aims to evaluate the efficacy and safety of neostigmine on treating patients with SAP.

The first defection time is a defined as the time span from the onset of disease to the first defection. Spontaneous defection is usually an indicator for starting EN in clinical practice and is widely used in researches on gastrointestinal obstruction [31–33]. One [34] research investigating transcutaneous electrical acustimulation for treating acute pancreatitis used first defection time as an important endpoint. In this study, we choose the first defection time as our primary outcome.

### Objectives {7}

The objective of this study is to investigate the efficacy and safety of neostigmine on treating SAP and to evaluate whether it can promote the recovery of intestinal function, making spontaneous defection and EN usage earlier, decrease intra-abdominal pressure, reduce mortality, organ failure and shorten hospital stay.

### Trial design {8}

**Fig. 1:**
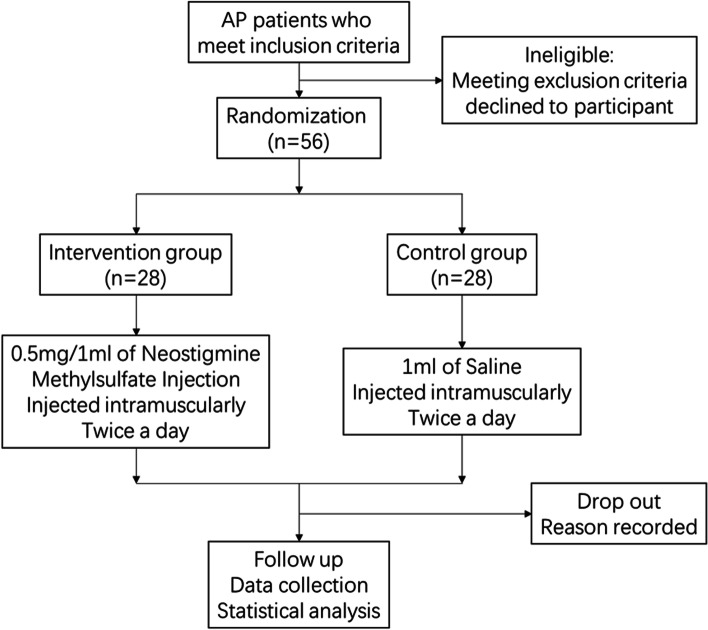
Flow chart of the study

This study is a randomized, placebo-controlled, double-blinded, mono-centric, superiority trial. All eligible participants will be randomly divided into intervention group or control group in a 1:1 ratio. Participants will be followed up until they are discharged. The flow chart of this study is shown in Fig. 1.

## Methods: participants, interventions, and outcomes

### Study setting {9}

The study will be conducted in the emergency department and intensive care unit (ICU) department of Peking Union Medical College Hospital (PUMCH), a grade III level A hospital in China.

### Eligibility criteria {10}

The detailed inclusion and exclusion criteria are outlined in Table 1. Briefly, patients with newly onset of acute alcoholic or hyperlipidemia AP and evidence of organ failure will be included. Patients that have contraindication to neostigmine methylsulfate injection or other severe diseases will be excluded.

**Table 1:** Eligibility criteria

Inclusion criteria	Exclusion criteria
• Aging between 18 and 75 • Diagnosed with alcoholic or hyperlipidemia acute pancreatitis • Evidence of organ failure (according to Marshall scoring system)	• Allergic to neostigmine methylsulfate injection • Contradiction to neostigmine methylsulfate injection ▪ Epilepsy ▪ Angina, arrhythmia, ventricular tachycardia, sinus bradycardia, ▪ Mechanical intestinal obstruction, urethra tract obstruction, asthma ▪ Uncontrolled hypotension, elevated vagus nerve tone • Pregnancy • Uncontrolled hypertension and severe cardiocerebrovascular disease

### Who will take informed consent? {26a}

Informed consent will be obtained by a member of study team.

For any AP patients who visit the emergency department of PUMCH and meets the inclusion and exclusion criteria, they will be approached to participate in the study by one of the investigators from the study team. The investigator will be responsible for providing each patient with an informed consent form (ICF) about the study’s purpose and procedures, foreseeable benefits and potential risks of participation, compensation for any potential harms, data protection procedures, and option to withdraw from the study at any time and without any given reason, which should be read by the patient. The investigator will answer any questions the patient may have, and both the patient and investigator will sign the ICF to indicate the patient’s full understanding of the protocol. Written informed consent must be obtained for all participants prior to any intervention.

### Additional consent provisions for collection and use of participant data and biological specimens {26b}

This trial does not involve collecting biological specimens. In the informed consent form, participants will be asked if they agree to use their data for further analysis and sharing with researchers relevant to this study should they choose to withdraw from the trial.

### Interventions

#### Explanation for the choice of comparators {6b}

The dose of neostigmine used in this trial (0.5 mg twice a day) has been chosen based on the guidance from FDA [35]. One milliliter of saline will be used as placebo, which will be taken identically.

#### Intervention description {11a}

Participants will be randomized in a 1:1 ratio via computer-generated sequence randomization. The study will be blinded to participants. Neostigmine methylsulfate injection and the saline are prepared by pharmacists who are not involved in the study and are identical visually.

For experiment group, 0.5 mg/1 ml of neostigmine methylsulfate injection will be injected intramuscularly at the deltoideus triangularis twice a day right after the enrollment. For control group, 1 ml of 0.9% NS will be injected intramuscularly at the deltoideus triangularis twice a day.

Apart from intervention, other treatment of AP will be performed according to American Gastroenterological Association Institute Guideline [36].

#### Criteria for discontinuing or modifying allocated interventions {11b}

Adverse events determined by clinical doctors will be reported to the study team and IRB. The study team will determine whether the event is likely to be due to intervention and whether to discontinue the study medication. The IRB will review the event routinely and determine whether it is safe to continue the study.

#### Strategies to improve adherence to interventions {11c}

All participants will be informed of the study procedures, as well as potential benefits and risks to make them fully understand the significance of their involvement in the study. The study team will check the nursing document of each participant routinely to determine that the participants are taking intervention properly.

#### Relevant concomitant care permitted or prohibited during the trial {11d}

There will be no restrictions regarding concomitant care during the trial.

#### Provisions for post-trial care {30}

Not applicable. Study participants will exit the study after discharging from hospital. There will be no further follow-up.

### Outcomes {12}

#### Primary outcome

The effect of neostigmine on gastrointestinal motility will be assessed using time span from onset of disease to the first time of spontaneous defection (defined as at least 1 h apart from enema). The defection times will be documented by clinical doctors and reported to study team for documentation.

#### Secondary outcome

- Mortality of patients
- New-onset organ failure, defined as organ failure after randomization (not present at any time before randomization). Including respiratory failure (PaO_2_/FiO_2_ ≦ 300, or requirement of mechanical ventilation), circulatory failure (systolic blood pressure < 90 mmHg, despite adequate fluid resuscitation, or requirement for inotropic catecholamine support) and renal failure (creatinine level > 177 μmol/L after rehydration or new need for hemofiltration or hemodialysis
- Intra-abdominal pressure, measured indirect using bladder pressure [37] every day after enrollment
- Timing of EN, defined as time from randomization to the initiation of tolerated EN
- Borborygmus, measured every day by member of study team
- Occurrence of abdominal infection, based on the diagnosis when discharging
- The proportion of adverse events and serious adverse events identified in both groups
- Health economics, including length of ICU stay, length of hospital study, and total cost in hospital, documented based on hospital information system

#### Participant timeline {13}

The participant timeline is presented in Table 2.

**Table 2:**
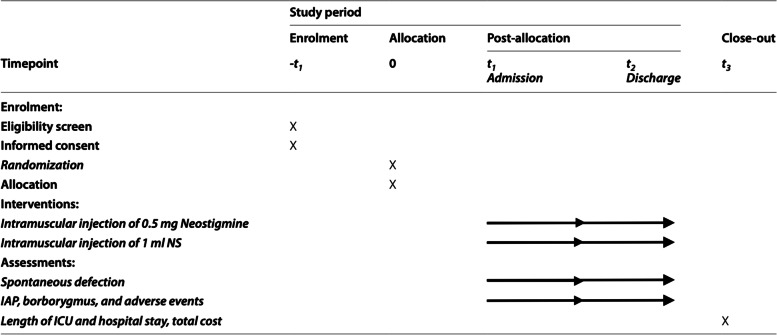
Participant timeline

#### Sample size {14}

This is a randomized controlled trial. The bilateral significance level is 5% with an assurance of 90%. We estimate the standard deviation is 36 h, and the mean difference is 36 h. In the case of 1:1 ratio between intervention group and control group, a total of 44 patients will be needed (22 patients in each group). Considering a 20% drop-out rate, 56 patients will be recruited.

#### Recruitment {15}

A total of 56 patients will be recruited and participate in the study. Prior to recruitment, investigators will carefully study the medical history, laboratory test, and imaging test of each patient. If the patient meets the inclusion and exclusion criteria, researchers will fully consider the compliance of the patients and informed consent forms (ICFs) will be obtained before collecting any data or performing any intervention. After initial assessment and randomization, patients will receive intervention and 48 h after onset. If the patient still has evidence of organ failure, they will be diagnosed with SAP and will be formally enrolled in the study. The follow-up will be ended when the patients leave the hospital.

### Assignment of interventions: allocation

#### Sequence generation {16a}

All participants will be randomly divided into the intervention group or the control group in a 1:1 ratio. Randomization will be performed using computer-generated sequence. No stratification is planned in this study.

#### Concealment mechanism {16b}

A computer program will be developed by technicians not related to the study. For each new participant entering the study, the investigator will open the program, and the participant will be assigned randomly. The program is blinded to individuals directly involved in the study.

#### Implementation {16c}

A computer program developed by technicians not involved in the assignment or care of the trial participants will be used to assign participants to intervention or control group. Study investigators will lead participant recruitment, informing participants of all the trial procedures and obtaining their ICF before the initial inclusion in the study. The study investigators will confirm the eligibility of the participant prior to any study procedures being undertaken.

### Assignment of interventions: blinding

#### Who will be blinded {17a}

The participants, clinical doctors, and outcome assessors will remain blinded to the intervention. However, data analysts will not be blinded.

#### Procedure for unblinding if needed {17b}

There should not be any need to unblind the participants. The investigation is not blinded to clinical doctors. Nevertheless, if required, unblinding can be carried out by the study team.

### Data collection and management

#### Plans for assessment and collection of outcomes {18a}

Sociodemographic characteristics and required clinical information will be obtained from the hospital information system of PUMCH. IAP and borborygmus of participants will be measured every day by the researcher. A third person with adequate clinical acumen independent to the project will assess the data collected. If inappropriate data is discovered, study team will be notified to remedy the error.

#### Plans to promote participant retention and complete follow-up {18b}

During study period, participants will stay in hospital. No follow-up after discharge is arranged. If there is a significant protocol violation, data will not be included in the analysis.

#### Data management {19}

The data will be collected in a Microsoft Excel database for assessment; data will be kept securely within the organization’s network. All data will be stored with the principal investigator in a password-protected computer for a period of 5 years. All soft copies of the data will be permanently deleted from the computer once the data storage time is over or at the conclusion of the study.

#### Confidentiality {27}

The collected information will remain anonymous; participants will be allocated a participant number for de-identification purposes. The data will be stored in a password-protected computer and will only be accessed by study team.

#### Plans for collection, laboratory evaluation, and storage of biological specimens for genetic or molecular analysis in this trial/future use {33}

Not applicable. No specimens will be collected.

### Statistical methods

#### Statistical methods for primary and secondary outcomes {20a}

1. Intention-to-treat (ITT): Nno one was excluded from the main analysis set after randomization
2. Per-protocol set (PPS): good compliance case data that meets the main inclusion and exclusion criteria and reach the primary endpoint

The number of participants selected and complete the study will be listed and the two analysis data sets (ITT and PPS) as specified above will be identified. Cases of protocol violation will also be listed with reasons documented. In terms of outcome indicator analysis, continuous variable indicators will count the number of cases (*n*), mean (*x*), standard deviation (SMD), median (*M*), minimum (min), and maximum (max). The difference between groups will be analyzed using Student’s *t*-test. Counting and grading data will be used to calculate the frequency and composition ratio. The *χ* ^2^ test/chi-square test will be used for differences between groups. Unless otherwise stated, all statistical tests will be conducted in a bilateral manner. All data will be entered in Microsoft Excel, and all statistical analyses will be performed using the SPSS version 25 statistical package.

#### Interim analyses {21b}

No planned interim analyses will be arranged because of relatively small sample size of this study.

#### Methods for additional analyses {20b}

Currently, there is no planned additional subgroup or adjusted analyses.

#### Methods in analysis to handle protocol non-adherence and any statistical methods to handle missing data {20c}

An ITT data set will be used for protocol non-adherence and no imputation of missing data will be performed for statistical analysis.

#### Plans to give access to the full protocol, participant level-data and statistical code {31c}

Any data required to support the protocol can be supplied on reasonable request. Only de-identified datasets will be supplied.

### Oversight and monitoring

#### Composition of the coordinating center and trial steering committee {5d}

This is a single-center trial. The study team will coordinate and manage the entire study process. A regular meeting will be arranged to discuss study questions of any type. An IRB consisting of three experts not involved in this study will be formed to monitor the trial.

#### Composition of the data monitoring committee, its role and reporting structure {21a}

This is a single-center trial with relatively small sample-size, data monitoring will therefore be conducted by study team. No data monitoring committee is required.

#### Adverse event reporting and harms {22}

All participants will be monitored with respect to any possible adverse events related to the administration of interventions. All adverse events (AEs) observed or reported by the participants or clinical doctors are collected and evaluated for relatedness to trial intervention, seriousness, severity, expectedness, and outcome by study team. All adverse events will be documented and reported to IRB.

#### Frequency and plans for auditing trial conduct {23}

For the quality assurance, 20% randomly selected data from the collected information will be re-interviewed. Any discrepancies will be corrected accordingly. In addition, the IRB will audit the data every month.

#### Plans for communicating important protocol amendments to relevant parties {25}

The principal investigator will be responsible for any protocol modification and any modification will be submitted to the Research Ethics committee of PUMCH for approval. The principal investigator will disseminate the changes of the protocol and all team members will be trained.

#### Dissemination plans {31a}

The study findings will be presented in national or international conferences. A full scientific article will be developed and published in peer-reviewed journal/s. The results of the study will be released to the participating patients.

## Discussion

Gastrointestinal dysmotility is a common complication in AP patients, which can lead to delayed feeding and OFI. There is little evidence regarding the treatment of gastrointestinal dysmotility induced by AP. Neostigmine can increase gastrointestinal motility and has been used to treat gastrointestinal dysmotility after surgery. With this randomized, placebo-controlled trial, our aim is to investigate whether neostigmine is safe and efficacy for treating AP patients with gastrointestinal dysmotility. We would like to discuss some aspects and challenges of the study design in detail below.

First, the enrollment of patients: a diagnosis of SAP requires persist organ failure, which is defined as organ failure lasting over 48 h [4]. However, early intervention [38] plays an important role in treating AP patients, so we start intervention on patients within 24 h of onset, which will lead to brief intervention on moderate-severe acute pancreatitis patients.

Another point we would to address is the COVID-19 pandemic, which may lead to fewer patients, disturbing patient enrollment and even temporary interruption of the research. A delayed end of this study may occur.

In conclusion, a positive outcome of this trial will provide strong evidence as to the safety and efficacy of neostigmine in treating the gastrointestinal dysmotility for AP patients.

### Trial status

The study is on its third version of the protocol and is expected to begin enrollment on May 1, 2022. Completion of the study protocol is expected to be near May 1, 2024, and the estimated end of study date will be September 1, 2024.

## Data Availability

All data are stored securely with the corresponding author on an organizational password-protected computer/network and will be destroyed 5 years after the study’s conclusion. The datasets analyzed during the study will be made available from the corresponding author on reasonable request. If the subjects are infringed due to data disclosure in the trial, the research team will provide them with appropriate compensation if necessary.

## Abbreviations

IRB: Institutional review board
AP: Acute pancreatitis
SAP: Severe acute pancreatitis
OFI: Oral feeding intolerance
ACS: Abdominal compartment syndrome
ACI: Acetylcholinesterase inhibitor
IAP: Intra-abdominal pressure
ICU: Intensive care unit
PUMCH: Peking Union Medical College Hospital
ICF: Informed consent form
ITT: Intention-to-treat
PPS: Per-protocol set
AE: Adverse event

## References

[1] Peery, A. F. et al. Burden of gastrointestinal, liver, and pancreatic diseases in the United States. Gastroenterology 149, 1731–1741. e1733 (2015). 10.1053/j.gastro.2015.08.045 PMC4663148 26327134

[2] Peery, A. F. et al. Burden and cost of gastrointestinal, liver, and pancreatic diseases in the United States: update 2018. Gastroenterology 156, 254–272. e211 (2019). 10.1053/j.gastro.2018.08.063 PMC6689327 30315778

[3] Peery AF (2022). Burden and cost of gastrointestinal, liver, and pancreatic diseases in the United States: update 2021. Gastroenterology.

[4] Banks PA (2013). Classification of acute pancreatitis—2012: revision of the Atlanta classification and definitions by international consensus. Gut.

[5] Koutroumpakis E (2017). Management and outcomes of acute pancreatitis patients over the last decade: a US tertiary-center experience. Pancreatology.

[6] Leppäniemi A (2019). 2019 WSES guidelines for the management of severe acute pancreatitis. World journal of emergency surgery.

[7] Wang X, Gong Z, Wu K, Wang B, Yuang Y (2003). Gastrointestinal dysmotility in patients with acute pancreatitis. J Gastroenterol Hepatol.

[8] Chapman MJ, Nguyen NQ, Deane AM (2013). Gastrointestinal dysmotility: evidence and clinical management. Curr Opin Clin Nutr Metab Care.

[9] Chen C-Y (1999). Endothelin-1 is a candidate mediating intestinal dysmotility in patients with acute pancreatitis. Dig Dis Sci.

[10] Windsor A (1998). Compared with parenteral nutrition, enteral feeding attenuates the acute phase response and improves disease severity in acute pancreatitis. Gut.

[11] Bakker OJ (2014). Early versus on-demand nasoenteric tube feeding in acute pancreatitis. N Engl J Med.

[12] Eckerwall GE, Tingstedt BB, Bergenzaun PE, Andersson RG (2007). Immediate oral feeding in patients with mild acute pancreatitis is safe and may accelerate recovery—a randomized clinical study. Clin Nutr.

[13] Zhao XL (2015). Early oral refeeding based on hunger in moderate and severe acute pancreatitis: a prospective controlled, randomized clinical trial. Nutrition.

[14] McKenzie, S. J. et al. The effect of enteral nutrition on adipokines in patients with acute pancreatitis. Journal of nutritional science 4 (2015). 10.1017/jns.2015.20 PMC4611076 26495124

[15] Bevan MG (2017). Incidence and predictors of oral feeding intolerance in acute pancreatitis: a systematic review, meta-analysis, and meta-regression. Clin Nutr.

[16] Chebli JMF (2005). Oral refeeding in patients with mild acute pancreatitis: prevalence and risk factors of relapsing abdominal pain. J Gastroenterol Hepatol.

[17] Levy P (1997). Frequency and risk factors of recurrent pain during refeeding in patients with acute pancreatitis: a multivariate multicentre prospective study of 116 patients. Gut.

[18] Whitlock, T. L. et al. Early readmission in acute pancreatitis: incidence and risk factors. Official journal of the American College of Gastroenterology| ACG 105, 2492–2497 (2010). 10.1038/ajg.2010.234 20531398

[19] Pendharkar SA (2015). Association between oral feeding intolerance and quality of life in acute pancreatitis: a prospective cohort study. Nutrition.

[20] Siebert M (2021). Management of abdominal compartment syndrome in acute pancreatitis. J Visc Surg.

[21] De Waele JJ, Leppäniemi AK (2009). Intra-abdominal hypertension in acute pancreatitis. World J Surg.

[22] Al-Bahrani AZ (2008). Clinical relevance of intra-abdominal hypertension in patients with severe acute pancreatitis. Pancreas.

[23] Boone B (2013). Abdominal compartment syndrome is an early, lethal complication of acute pancreatitis. Am Surg.

[24] Chen H, Li F, Sun J-B, Jia J-G (2008). Abdominal compartment syndrome in patients with severe acute pancreatitis in early stage. World J Gastroenterol: WJG.

[25] Parthasarathy G (2015). Effect of neostigmine on gastroduodenal motility in patients with suspected gastrointestinal motility disorders. Neurogastroenterol Motil.

[26] Ravi K (2010). Phenotypic variation of colonic motor functions in chronic constipation. Gastroenterology.

[27] Caldarella MP, Serra J, Azpiroz F, Malagelada JR (2002). Prokinetic effects in patients with intestinal gas retention. Gastroenterology.

[28] Liao Y, Li Y, Ouyang W (2021). Effects and safety of neostigmine for postoperative recovery of gastrointestinal function: a systematic review and meta-analysis. Annals of Palliative Medicine.

[29] Ponec RJ, Saunders MD, Kimmey MB (1999). Neostigmine for the treatment of acute colonic pseudo-obstruction. N Engl J Med.

[30] He W (2022). Randomized controlled trial: neostigmine for intra-abdominal hypertension in acute pancreatitis. Crit Care.

[31] Li, Z., Liu, Z. & Yu, Z. Application effect of somatostatin combined with transnasal ileus catheterization in patients with acute intestinal obstruction and advanced gastric cancer. Computational Intelligence and Neuroscience 2022 (2022). 10.1155/2022/9747880 PMC9206574 35726291

[32] Hasler-Gehrer S (2019). Does coffee intake reduce postoperative ileus after laparoscopic elective colorectal surgery? A prospective, randomized controlled study: the coffee study. Dis Colon Rectum.

[33] Lu Y (2021). Effect of intraoperative dexmedetomidine on recovery of gastrointestinal function after abdominal surgery in older adults: a randomized clinical trial. JAMA Netw Open.

[34] Xuan, J. l. et al. Integrative effects of transcutaneous electrical acustimulation on abdominal pain, gastrointestinal motility, and inflammation in patients with early-stage acute pancreatitis. Neurogastroenterology & Motility 34, e14249 (2022). 10.1111/nmo.14249 34536258

[35] FDA. Summary reviews: (https://www.accessdata.fda.gov/drugsatfda_docs/nda/2013/204078Orig1s000TOC.cfm)

[36] Crockett SD (2018). American Gastroenterological Association Institute guideline on initial management of acute pancreatitis. Gastroenterology.

[37] Milanesi R, Caregnato RCA (2016). Intra-abdominal pressure: an integrative review Einstein (Sao Paulo).

[38] James TW, Crockett SD (2018). Management of acute pancreatitis in the first 72 hours. Curr Opin Gastroenterol.

